# Research Progress in High-Efficiency Utilization of Nitrogen in Rapeseed

**DOI:** 10.3390/ijms24097752

**Published:** 2023-04-24

**Authors:** Na Zhan, Kun Xu, Gaoxiang Ji, Guixin Yan, Biyun Chen, Xiaoming Wu, Guangqin Cai

**Affiliations:** Key Laboratory of Biology and Genetic Improvement of Oil Crops, Ministry of Agriculture and Rural Affairs, Oil Crop Research Institute, Chinese Academy of Agricultural Sciences, Wuhan 430062, China

**Keywords:** rapeseed, nitrogen-efficient germplasm, physiological basis, molecular mechanism

## Abstract

Nitrogen (N) is one of the most important mineral elements for plant growth and development and a key factor for improving crop yield. Rapeseed, *Brassica napus*, is the largest oil crop in China, producing more than 50% of the domestic vegetable oil. However, high N fertilizer input with low utilization efficiency not only increases the production cost but also causes serious environmental pollution. Therefore, the breeding of rapeseed with high N efficiency is of great strategic significance to ensure the security of grain and oil and the sustainable development of the rapeseed industry. In order to provide reference for genetic improvement of rapeseed N-efficient utilization, in this article, we mainly reviewed the recent research progress of rapeseed N efficiency, including rapeseed N efficiency evaluation, N-efficient germplasm screening, and N-efficient physiological and molecular genetic mechanisms.

## 1. Introduction

Nitrogen (N) is one of the essential elements required for plant growth and development and is an important component of chlorophyll, nucleic acid, protein, alkaloids, and other secondary metabolites [1,2]. It is also one of the main factors affecting crop yield and quality in production [3]. The massive application of N fertilizer has significantly increased crop yield in China. However, with the intensive planting of modern agricultural production, the N source that can be directly absorbed and utilized in cultivated soil is increasingly limited. In order to meet the food demand, the input of N fertilizer increases sharply [4,5]. Moreover, the excessive fertilization by famers is quite common under the influence of the concept of “high input is equal to high yield” in China. According to statistics, China’s fertilizer application accounts for one-third of the world’s total fertilizer production, ranking the first in the world, of which N fertilizer accounts for about 60%, while the N fertilizer utilization rate of farmland is only 40.2%, far below the world average level [6,7]. The converted amount of agricultural N fertilizer applied in 2017 was 22.2181 million tons, an increase of nearly 1.37 times compared with 1980, while the grain yield only increased by about 1.06 times (National Bureau of Statistics data of the People’s Republic of China, https://data.stats.gov.cn/ (accessed on 20 November 2022)). Therefore, excessive N fertilizer input does not bring high grain output, which not only increases the agricultural production cost and leads to the decline of economic benefits but also causes different degrees of environmental damage and affects the sustainable development of agricultural ecology. Most of the N fertilizer that cannot be absorbed by plants accumulates in soil and enters different water bodies such as rivers, seas, and lakes through soil erosion, which results in eutrophication of the water body [8]. There are also some fertilizers volatilized into the atmosphere in the form of nitrogen oxides and ammonia, causing air pollution [5]. Therefore, the way in which to improve N fertilizer use efficiency, reduce the application of N fertilizer, and ensure grain yield is the key issue to be solved urgently in agricultural development.

**Figure 1:**
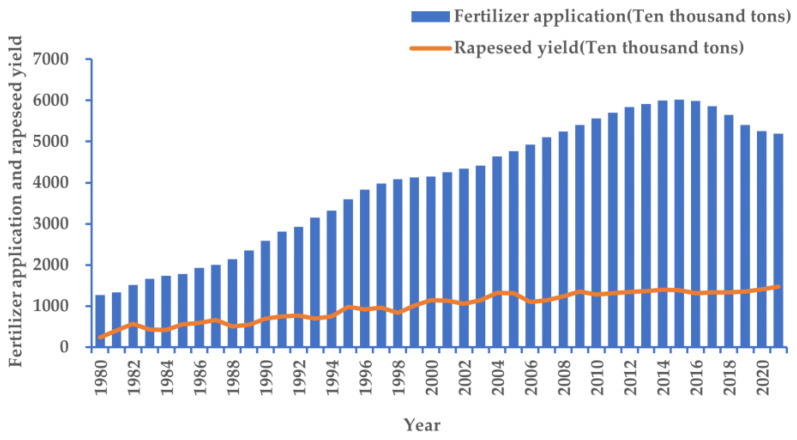
The correlation between rapeseed yield and fertilizer application in China in previous years. (The data come from the National Bureau of Statistics of the People’s Republic of China.)

Rapeseed is the third most important vegetable oil source worldwide, behind palm and soybean [9]. For a long time, the planting area of rapeseed has maintained a stable and slightly increased trend in most parts of the world. According to statistics, the global sown area of rapeseed increased from 33.427 million hm^2^ to 36.107 million hm^2^ during 2016–2020 (United States Department of Agriculture. Foreign agricultural service. https://apps.fas.usda.gov/psdonline/app/index.html#/app/advQuery (accessed on 22 November 2022)). Rapeseed is the main oil crop in China. Since the self-sufficiency rate of edible oil is declining, while the dependency on foreign countries is increasing in China, vigorously developing rapeseed production is of great strategic significance to maintaining the security of the national edible oil supply. Rapeseed has a long history of cultivation in China. The main cultivation types are *Brassica rapa*, *Brassica juncea*, and *Brassica napus*. Among them, *Brassica napus* (hereinafter referred to as rapeseed) accounts for about 90% of the total planting area of rapeseed in China. Compared with other crops, rapeseed requires high N fertilization during growth [10]. Although rapeseed has considerable N absorption capacity, it is usually described as a crop with low N utilization rate, which is about half of that of cereal crops [11,12]. According to the data of the National Bureau of Statistics, the growth of rapeseed production in China was almost at a standstill during the decade from 2004 to 2015, but the application of fertilizer has been on the rise, from 12.694 million tons in 2004 to 60.226 million tons in 2015, an increase of nearly 3.74 times. In 2016, the Ministry of Agriculture and Rural Affairs organized a “double reduction action” on the use of chemical fertilizers and pesticides. As a result, the use of chemical fertilizer has declined since 2016, but there was no significant change in rapeseed production (Figure 1). This shows that the low nitrogen use efficiency (NUE) has become one of the important factors limiting the yield improvement of rapeseed in China in recent years [13]. It caused serious water pollution and eutrophication in the Yangtze River Basin. Therefore, it is urgent to improve the NUE of rapeseed by studying its nutrient efficiency, so as to provide new ideas, methods, and technical paths to break through the bottleneck of rapeseed yield improvement, as well as to provide a paradigm for a new model of resource-friendly and environment-saving agricultural production.

The methods to improve the NUE of rapeseed mainly include the following: (1) Screening the excellent germplasm for high NUE, mining the key genes for high NUE, and then selecting and breeding high NUE varieties to improve the NUE of rapeseed. (2) Precise fertilization. The way of spreading or the process of surface fertilization and excessive N fertilizer dosage of Chinese farmers can easily cause fertilizer volatilization and loss, which are not conducive to crop absorption and thus reduce NUE. Therefore, it is necessary to strengthen the research on precise fertilization technologies to form a simple and feasible technical system, such as precise management of farmland nutrients, precise fertilization technology, and new fertilizer technology, among which new fertilizer technologies include the CULTAN (controlled uptake long term ammonium nutrition) fertilizer method integrating N fertilizer form, fertilization method, and fertilization period [14], as well as the application of fertilizers containing urease inhibitors or nitrification inhibitors, which can extend the use time of fertilizers by slowing down urea hydrolysis and N nitrification. (3) Balanced fertilization. The key to improve fertilizer use efficiency is to ensure the comprehensive and balanced supply of various macro- and micronutrient elements necessary for crops. For example, the utilization of soil N by plants is closely related to soil sulfur content. Studies have shown that excessive N fertilizer application under the condition of insufficient sulfur fertilizer supply will lead to crop nutrient imbalance, thereby limiting protein synthesis and reducing rapeseed yield. For soils with severe sulfur deficiency, the application of N fertilizer will aggravate the soil sulfur deficiency, resulting in reduced crop yield [15]. According to statistics, the average soil content of available sulfur in the Yangtze River Basin, the main producing area of rapeseed in China, is 22.6 mg·kg^−1^, which is in a potential deficiency state [16]. Similarly, molybdenum, a trace element necessary for plants, is also related to the NUE of rapeseed. Molybdenum is the active component of nitrate reductase. When molybdenum is deficient, the activity of nitrate reductase decreases, affecting the plants’ N assimilation process. Moreover, there is a molybdenum deficiency phenomenon in the Yangtze River Basin, and the soil effective molybdenum content is below 0.15 mg·kg^−1^ [17]. Therefore, when applying N fertilizer, various macro- and micronutrients should be supplied to crops in scientific proportions according to the soil nutrient content. (4) According to the precipitation year type to determine the optimal rapeseed N application amount and period. A study found that the concentration of total N loss in rainfall runoff would increase with the increase in rainfall, and compared with dry years, the nitrate N leaching amount increased in years with abundance of water, while the Yangtze River Basin has abundant precipitation resources, and the total precipitation of rapeseed during the whole growth period was 680 mm on average, showing an increasing trend year by year [18]. Therefore, the amount and period of N application should be adjusted according to rainfall and precipitation period, so as to slow down the leaching of soil N and improve the NUE of rapeseed while ensuring the yield. (5) Adopting modern biological technology to improve the N efficiency of rapeseed, for instance, utilizing transgenic methods to improve the related genes of N absorption, transport, and assimilation, so as to improve its utilization efficiency. Although rapeseed NUE can be improved from the above different levels, the NUE of rapeseed depends to a greater extent on its own genetic characteristics. Therefore, it is the most economical and effective way to fundamentally solve rapeseed low NUE by screening rapeseed germplasm with high NUE, exploring its genetic potential of N nutrition through biological approaches, improving its N efficiency by genetic improvement, and breeding rapeseed varieties with high NUE.

This paper summarizes the research progress of efficient use of N in rapeseed, which includes N efficiency evaluation, germplasm screening of efficient use of N, and physiological and molecular genetic mechanism of efficient use of N. Our review provides a reference for the genetic improvement of the efficient use of N in rapeseed.

## 2. Evaluation of Nitrogen Efficiency and Screening of Nitrogen-Efficient Germplasm

### 2.1. Definition and Evaluation of Nitrogen Efficiency

Nitrogen efficiency is the ability of plants to absorb and utilize N, and it is usually expressed in terms of the ratio of N input to output per unit. Plant N efficiency is subjected to a combination of genetic and environmental factors. It involves uptake, transport, assimilation, utilization, and reuse of N by plants [19]. As early as 1939, Harvey discovered the differences in N uptake and utilization among different maize varieties and proposed the concept of “nitrogen efficiency genotype difference”. 

Although many studies have been performed on the differences of N efficiency in different crops, there is still no unified standard for the definition and evaluation index of plant N efficiency. One of the most accepted and affirmed definitions of N efficiency by researchers is the grain yield produced per unit of available N, which is equal to the product of N absorption efficiency and N use efficiency. N absorption efficiency is the ratio of total N absorbed during the whole growth period of the plant to N supply. N use efficiency represents the dry matter mass or yield produced by unit N absorption in plants [20,21]. It has not been reported as to which contributes more to plant N efficiency. Some studies have shown that changes in N efficiency under low N conditions are mainly caused by differences in N absorption efficiency, while differences in N use efficiency under high N conditions are more important in plants [22]. Worku [23] believed that both N absorption efficiency and utilization efficiency are equally important under any N level. From the perspective of agricultural production practice, the efficient use of N by crops is mainly manifested in the following two ways: Firstly, crops can obtain higher yield when N supply is insufficient. Secondly, crop yield can also increase with the increase in N supply [24]. Bouchet [9] divided rapeseed N efficiency into the following four types on the basis of yield: (1) Inefficient and non-responsive, that is, rapeseed yield is low regardless of the N supply level. (2) Inefficient and responsive, that is, yield is high under high N conditions and yield is low under low N conditions. (3) Efficient and responsive, that is, yield is high at any N level. (4) Efficient and non-responsive, that is, yield is excellent under low N conditions and average yield under high N conditions.

### 2.2. Screening of High Nitrogen Efficiency Rapeseed Germplasm

The N requirements of different crops vary greatly. Among food crops, rice requires approximately 2.0–2.4 kg of N per 100 kg grain, while dry crops such as wheat and maize require 3.0–4.0 kg of N. Soybean belongs to the Fabaceae family with high N requirement, which is about twice that of cereal crops. For every 100 kg of soybean seeds produced, 8.3 kg of pure N needs to be absorbed, but they can obtain N from the atmosphere through symbiotic N fixation with rhizobia, which is about 40–60% of the N requirement of soybean. Compared with soybean, rapeseed requires more N, 8.8–11.3 kg of N absorbed for per 100 kg seed produced (Agricultural planting network, https://www.my478.com (accessed on 15 February 2023)). N efficiency varies not only among different species but also among different varieties of the same species. For example, there are generally large differences in N efficiency between indica and japonica subspecies of rice [25], with indica usually showing better nitrate utilization ability [26,27]. Inthapanya P. et al. [28] suggested that NUE is affected slightly by the interaction between genes and the environment, while genotypes of the germplasm play a decisive role in NUE. The NUE of germplasms with different genotypes could remain relatively stable over time. Therefore, it is completely feasible to select scientific evaluation indexes and reasonable screening methods to obtain efficient N genotypes. 

Over the years, researchers have carried out much research work in the evaluation and identification of the nutrient-efficient germplasm of crops such as *Oryza sativa*, *Triticum aestivum*, *Zea mays*, *Gossypium hirsutum*, and *Setaria italica*. It provides us with a reference for the screening of rapeseed N-efficient germplasm. Currently, the studies on the selection of N-efficient germplasms of different crops mainly include screening at the seedling stage and the whole growth stage. Due to the long screening cycle of the whole growth stage, it is susceptible to environmental influences and cannot truly reflect the genetic potential of low-nitrogen tolerance traits of crops. Thus, more studies are conducted at the seedling stage for the primary screening of crops and then are verified at the whole growth period stage [29]. The screening methods of N-efficient germplasm include the field and pot simulation experiment, where the latter can be divided into the soil culture test, sand culture test, and hydroculture test method according to different media. The field experiment is the screening method closest to the production reality. However, its test environment is complicated and susceptible to other factors during the growth period, and thus it is difficult to control the test conditions for accurate screening. The soil culture experiment is also close to the actual production, but it is difficult to study the root morphology and related physiological and biochemical characteristics. The hydroponics test can accurately control the content of various components, which is evenly distributed in the nutrient solution. However, the lack of air in the culture environment can easily cause the change of nutrient solution concentrations. The sand culture test is a research method that can simplify the chemical state of the medium and concentrate on a certain component, but its medium has a poor chemical buffer capacity [29]. Therefore, researchers usually use different screening methods or a combination of two methods for germplasm screening. Zhai et al. used a combination of hydroponics and field experiments to evaluate the N efficiency of 14 rice germplasms at seedling, tillering, and maturity under different N concentration conditions and used four seedling traits (root tips number, root to shoot ratio, root dry mass, and shoot dry mass) and two maturity traits (effective panicle number and yield per plant) as the main evaluation indexes to screen low-N-tolerant rice varieties [30]. Yuan et al. used hydroponic test methods to evaluate the N efficiency of 78 soybean germplasms at the seedling stage under the two treatment levels of normal N (7.5 mmol/L) and low N (0.75 mmol/L), and six indexes, namely, total dry weight, whole plant N content, total N of whole plant, total root length, root volume, and root surface area, were used as the key indicators for the evaluation of low N tolerance at the seedling stage of soybean, with a pair of extremely different germplasms with N efficiency identified accordingly [31]. Gu et al. [32] carried out a study of the biological traits and N accumulation of 162 rapeseed germplasms under low N (237.5 μmol/L) and normal N (9500 μmol/L) conditions by nutrient solution culture and concluded that biomass could be used as the main index to evaluate N efficiency at the seedling stage. Taking the average plant biomass of different nitrogen levels as the threshold, 162 rapeseed germplasms were preliminarily divided into 3 categories according to their N efficiency, namely, 23 double efficient type, 28 double inefficient type, and 111 intermediate type. In summary, the evaluation of N efficiency can be combined with the performance of seedling stage and the whole growth stage, and the comprehensive use of the field test and pot simulation test can ensure the reliability of the test results.

## 3. Physiological Basis of Nitrogen-Efficient Rapeseed

Nitrogen efficiency is an intrinsic complex trait that is composed of grading processes of N absorption, transport, assimilation, and following performances of growth and development [19]. These processes are closely linked and interact with each other and are realized through a series of physiological and biochemical processes in plants.

### 3.1. Root System Characteristics

The root system is not only an important organ for plants to absorb water and nutrients but also can synthesize a variety of physiologically active substances. It is the first organ to sense changes in rhizosphere environmental conditions and show strong plasticity when the external environment changes [33]. Root morphological and physiological characteristics have important effects on the growth and development of shoots, yield, and quality [34,35]. Soil N is the main source of plant N, so the ability of roots to absorb and transport N plays a decisive role in plant N absorption efficiency to a certain extent [36]. Studies have shown that the root system of N-efficient rapeseed varieties in the vegetative growth stage grows more rapidly than that in the aboveground part in order to obtain more N for their growth and development [37,38]. Kamh et al. [37] compared two varieties of *B. napus* with different genotypes and found that the high-yielding varieties had larger root growth under a low N environment. Zou et al. [39] studied the root morphology of six rapeseed varieties with significantly different N efficiency under low N conditions at bolting stage, and the result showed that three N-efficient genotypes were higher than the three N-inefficient genotypes in root length, root surface area, root volume, root diameter, and root dry weight. Ye et al. [40] studied N use of two different genotypes in a nutrient solution of *B. napus* and found that the root volume and root active absorption area of N-efficient genotypes were larger than N-inefficient genotypes. Similarly, Wang et al. [41] took two rapeseed germplasms with contrasting N efficiency as materials to study the changes and differences of root morphology at seedling stage under low N stress by the hydroponic method. The results showed that N-efficient rapeseed had stronger tolerance to low N stress, a more developed root system, and a stronger ability to absorb and accumulate nutrients compared with N-inefficient germplasm. Therefore, it can be inferred that N-efficient rapeseed germplasms can maintain a high N efficiency under low N stress by maintaining a developed root system to improve the nutrient uptake and accumulation capacity.

### 3.2. Enzyme Activities Related to Nitrogen Metabolism

Inorganic N in soil usually exists in the form of nitrate and ammonium, which are the main N sources that can be absorbed and utilized by plants [42,43]. Moreover, both of them can be further utilized by plants only after a series of assimilation processes. Most NO_3_^−^ absorbed by plant roots is transported to the aboveground part for assimilation and utilization. First, NO_3_^−^ is reduced to NO_2_^−^ in the cytoplasm by nitrate reductase (NR), and then NO_2_^−^ is transported to the plastid, being reduced to NH_4_^+^ by nitrite reductase (NiR) to enter the ammonium assimilation process [44,45,46]. Finally, NH_4_^+^ enters the glutamate cycle under the action of glutamine synthetase/glutamate synthetase (GS/GOGAT). In this cycle, NH_4_^+^ interacts with glutamate to produce glutamine under the catalysis of GS, and then glutamine with α-ketoglutaric acid in the action of GOGAT again produce glutamate [47]. The activities of these key enzymes in plant N metabolism vary in terns of genotype between different species, different varieties of the same species, and different organs, which lead to different N metabolism rates of plants and thus lead to differences in plant NUE [48].

NR is the first enzyme that plants absorb NO_3_^−^, and it is also the rate-limiting enzyme in the process of nitrate assimilation. The activity of NR can reflect the N nutrition status and metabolism level of plants. Studies have shown that there are significant differences in genotypes and N levels in rapeseed NR activity [49]. Compared with N-inefficient genotypes, N-efficient genotypes have higher NR activity in leaves [50]. Zou et al. [39] found that NR activity in leaves and roots of N-efficient genotypes was higher than that of N-inefficient genotypes, and NR activity in leaves of rapeseed with the same genotype was higher than that in roots. Wang et al. [51] studied NR activity of rapeseed genotypes with different N efficiency at different N levels. The result showed that NR activity in leaves and roots of N-efficient genotypes was significantly higher than that of N-inefficient genotypes under low N stress. However, under normal N supply conditions, NR activity in leaves with different N efficiency genotypes had no difference, while NR activity in roots still had a significant difference. The results of Li et al. [52] showed that NR activity in shoots and roots of rapeseed N-efficient genotypes was higher than that of N-inefficient genotypes under low N stress, which was consistent with previous studies, indicating that N-efficient genotypes has a strong NO_3_^−^ assimilation ability.

GS is the main enzyme involved in ammonia assimilation and glutamine formation, playing an important role in N nutrition. Previous studies have shown that N level has a great effect on GS activity. At a normal N level, GS activity in leaves and roots of rapeseed were significantly higher than those under a low N level, and under both low N stress and normal N conditions, GS activity in leaves of high N efficiency germplasm was higher than that of low N efficiency germplasm, but the difference was more significant under low N stress [39,40,51,52]. The above result shows that the N-efficient germplasm also has a strong ammonium N assimilation ability, and the leaf has a stronger ability to assimilate ammonium N than the root system.

### 3.3. Photosynthesis

Photosynthesis is the main method of dry matter formation in plants. N is an important component of chlorophyll and Rubisco, and both the content of Rubisco and chlorophyll are highly correlated with N of leaf. Increasing leaf N content will significantly increase the photosynthetic rate [53,54]. SPAD value represents the relative value of chlorophyll content in plant leaves, which has a good correlation with the photosynthetic rate [55]. Therefore, this value can reflect the N nutrition of plants and the intensity of leaf photosynthesis to a certain extent. Wang et al. [56] showed that the SPAD value of the lower leaves of rapeseed germplasm with high N efficiency under low N treatment was significantly higher than that of rapeseed germplasm with low N efficiency, indicating that the former could maintain photosynthesis for a longer time and then synthesize more photosynthates under the condition of N deficiency. Han et al. [57] analyzed photosynthetic parameters of rapeseed with different N efficiency and found that chlorophyll concentration, photosynthetic rate, and intercellular CO_2_ concentration were significantly lower in N-inefficient genotypes than N-efficient genotypes at both seedling and flowering stages. Li et al. [52] also found that the photosynthetic rate and Rubisco activity of normal N supplying plants were higher than those of N-deficient plants, and the photosynthetic rate and Rubisco activity of the N-inefficient germplasm were lower than those of the N-efficient germplasm under any N supply level. Therefore, a higher photosynthetic rate may also be one of the physiological mechanisms of N-efficient rapeseed.

## 4. Molecular Basis of Nitrogen-Efficient Rapeseed

The process of a plant absorbing N from soil is mainly realized by its internal transporters. The N absorbed still needs to complete the whole N metabolism process undergoing a series of assimilation actions under the action of different enzymes in the plant. In recent years, researchers have isolated or cloned nitrate and ammonium transporter genes [58,59], genes encoding plant N metabolism enzymes [60,61,62], and some transcription factors related to plant N response [63,64,65] through molecular biological means.

### 4.1. Nitrogen Absorption and Transport Genes

Nitrate uptake by plants in soil is mainly mediated by nitrate transporters 1 (NRT1) and nitrate transporters 2 (NRT2). According to their nitrate transport activity, they can be divided into high- and low-affinity groups. When the external nitrate concentration is higher than 1 mmol·L^−1^, the NRT1 protein (except NRT1.1) mainly plays the role of a low-affinity nitrate transporter, and when the nitrate concentration is lower than 1 mmol·L^−1^, the NRT2 protein mainly plays the role of high-affinity nitrate transporter [66,67,68]. NRT1 is also known as NPF (nitrate transporter 1/peptide transporter family) [69], and the absorption process of ammonium nitrogen is regulated by ammonium transporter (AMT) [70].

**Figure 2:**
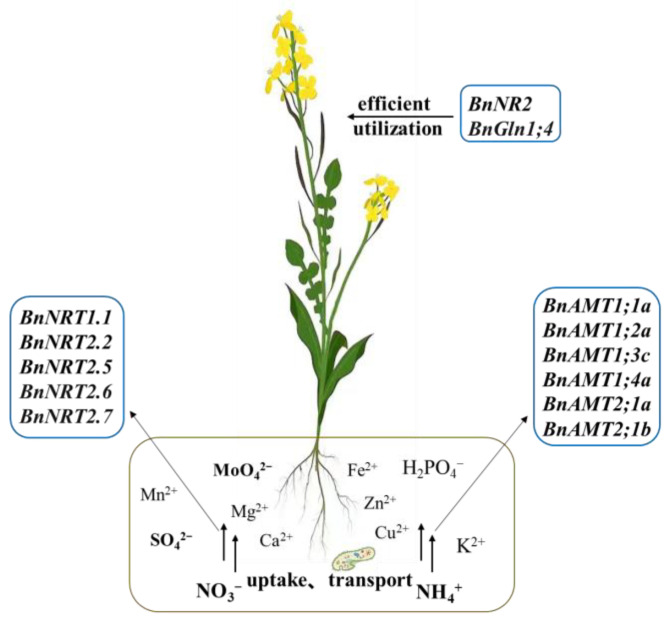
Diagram of the possible pattern of rapeseed N efficiency. (Note: The brown box represents the soil, which contains other elements and microorganism that affect nitrogen uptake by plants.)

At present, some nitrate and ammonium transporter genes related to N efficiency have been found in rapeseed. Wang et al. [51,56] found that under low N conditions, the expression levels of *BnAMT1;1*, *BnNRT1.1*, *BnNRT2.5*, *BnNRT2.6*, *BnNRT2.7*, and *BnNRT3* in the roots of N-efficient genotypes were significantly higher than those of N-inefficient genotypes. Moreover, low N induced the expression of *BnAMT1;1*, *BnNRT1.1*, *BnNRT2.5*, *BnNRT2.6*, and *BnNRT2.7*. Han et al. [57] showed that the expression level of the *BnNRT1.5* gene involved in xylem NO_3_^−^ loading was significantly upregulated in N-efficient genotypes compared with N-inefficient genotypes. However, the expression level of the *BnNRT1.8* gene involved in xylem NO_3_^−^ unloading showed a completely opposite trend to *BnNRT1.5* in the two genotypes. Chao et al. [71] used real-time quantitative PCR to study and analyze the differential expression of genes related to N uptake and transport (*BnNPFs*, *BnNRT2s*, and *BnAMTs*) in rapeseed germplasm with different N efficiencies. The results showed that the relative expressions of genes involved in NO_3_^−^ and NH_4_^+^ uptake and transport in N-efficient rapeseed germplasm were significantly higher than those in N-inefficient rapeseed germplasm under normal N supply conditions. The expression levels of genes *BnNRT2.4a*, *BnNRT2.5a*, and *BnNRT2.5b* related to NO_3_^−^ uptake and transport in roots were significantly lower in the N-efficient germplasm than in N-inefficient germplasm under low N stress. However, the *BnNPF7.3a* (*BnNRT1.5*) and *BnNPF6.2c* (*BnNRT1.4*) genes associated with NO_3_^−^ transport and redistribution, as well as the *BnAMT1;1a*, *BnAMT1;2a*, *BnAMT1;3c*, *BnAMT1;4a*, *BnAMT2;1a*, and *BnAMT2;1b* genes involved in regulating NH_4_^+^ uptake and transport were significantly higher than that of N-inefficient rapeseed germplasm. The results of the expression levels of the NO_3_^−^ transporter gene in different N efficiency genotypes were different from previous studies, which may be related to various factors such as the test materials, sampling period, and screening pressure, as well as the need to be further studied and verified. In summary, the above studies indicated that the higher N uptake efficiency of the N-efficient genotypes may be related to the high expression levels of some *BnNRTs* and *BnAMTs* genes (Figure 2).

### 4.2. Genes Related to Nitrogen Metabolism

Besides N transporter genes, the genes encoding enzymes related to N metabolism also play an important role in the process of efficient N absorption in rapeseed (Table 1). For example, Orsel et al. [72] found a total of 16 *BnGln1* genes, including 2 *BnGln1;1*, 2 *BnGln1;2*, 6 *BnGln1;3*, 4 *BnGln1;4*, and 2 *BnGln1;5*, in the rapeseed genome, which mainly encode glutamate synthetase in the cytoplasm. Among them, *BnGln1;1* and *BnGln1;4* families can respond to low N stress, and *BnGln1;2* family expression is upregulated under high N conditions. Wang et al. [51] used hydroponic culture to study the expression differences of genes encoding NR and GS with different N efficiency at the seedling stage in the rapeseed germplasm. The results showed that the expression levels of most members of the NR and GS gene families were significantly downregulated under low N stress, but the expression degree was different in different nitrogen efficiency genotypes. For example, under normal N supply conditions, the expressions of *BnGln1;1*, *BnGln1;2*, and *BnGln1;4* in the roots of N-efficient genotypes were significantly higher than those of N-inefficient genotypes, whereas the expression levels of these genes showed an opposite trend under low N treatment. In addition, low N stress also induced the expressions of *BnGln1.1* and *BnGln1;4* in N-efficient leaves, suggesting that they may play an important role in the N assimilation process of N-efficient genotypes. As for the genes encoding NR, this study found that under different N supply conditions, the expression level of *BnNR1* in the leaves and roots of N-efficient genotypes was significantly lower than that of N-inefficient genotypes. However, under low N conditions, the expression level of *BnNR2* in the leaves and roots of N-efficient genotypes was significantly higher than that of N-inefficient genotypes. Xiao et al. [73] detected 19 *BnGlns* family genes in the whole genome of rapeseed, for which expression profile analysis showed that the expression level of *BnGlns* family genes was generally upregulated after low N treatment. For example, in the *BnGln1.1s* subfamily, the expression level of *BnA4Gln1;1* in roots was significantly increased. The expression levels of some genes of the *BnGln1;4s* subfamily also showed an increasing trend in roots. In conclusion, N-efficient rapeseed germplasm was shown to have a stronger reduction, assimilation, and metabolic capacity for N absorbed into the body compared with the N-inefficient germplasm, which might be related to the high expression levels of *BnNR2* and *BnGln1;4*.

### 4.3. Transcription Factors

Transcription factors are a class of protein molecules that can recognize specific motifs of upstream promoters of genes and regulate gene expression [74]. They play a very important role in plant life activities and can adapt to various stressful environments by regulating the expression of relevant genes [75,76,77,78]. For example, NIA and WRKY play an important role in the regulatory process of NR and the response process of growth, development, and metabolism in plants [79]. Xiao et al. [73] found that the expression of most genes in the *BnNIAs* family was upregulated after low N treatment in rapeseed. They also analyzed the transcription factors WRKYs and MYBs, and the results showed that the gene expressions of *BnWRKY33s* and *BnWRKY70s* decreased after low N stress in rapeseed roots. The expression of most genes in *BnMYB4s*, *BnMYB44s*, and *BnMYB51s* subfamily was also decreased in the root system. Therefore, rapeseed can adapt to low N stress by regulating the expression levels of *BnaNIAs*, *BnaWRKYs*, and *BnaMYBs* genes.

## 5. Problems and Prospects

Rapeseed is not only the largest domestic vegetable oil source but also the second largest source of feeding protein, with an annual output of about 8 million tons of high-protein feeding meal (China Agricultural Information Network, http://www.agri.cn/province/henan/dsxxlb/202206/t20220609_7860848.htm (accessed on 21 December 2022)). Compared with other crops, rapeseed requires more N fertilizer input and less utilization efficiency during its growth period. More than half of the N fertilizer is lost to the environment [80], which increases agricultural production costs and causes serious environmental pollution. Currently, low NUE has become one of the important limiting factors for the increase in rapeseed yield. Therefore, the way in which to improve the efficiency of N utilization, reduce the application of N fertilizer, and realize the increase in rapeseed yield are the scientific problems that need to be solved. The studies on the mechanism of N-efficient utilization in rapeseed mainly focus on the physiological mechanism, and the molecular mechanism lacks further exploration. Although some genes related to N uptake, transport, and assimilation have been found, the way in which these genes perceive changes in N sources and are regulated by factors remains unknown, and further studies are needed. In the future, we can focus on the following points:

(1) Establishing an evaluation and identification system for rapeseed germplasm with high N efficiency. Although many studies have been conducted on the screening of rapeseed N-efficient germplasm, there is still no consensus on the evaluation indexes of low N tolerance, which may lead to different N efficiency evaluation results for the same rapeseed germplasm. Therefore, it is necessary to deeply study the relationship between morphological structure and N efficiency to find the most appropriate screening index and establish a set of scientific and reasonable evaluation and identification methods for N-efficient genotypes in rapeseed, so as to fully tap the genetic potential of rapeseed N efficiency and lay a foundation for improving its NUE through biological approaches.
(2) Using a multi-omics technique to comprehensively analyze the biological mechanism of N efficiency in rapeseed. The process of efficient N uptake and utilization is a very complicated process that is affected by many factors such as physiological level, developmental process, and external environment. It is difficult to fully describe the process using a single omics technique. Therefore, a natural population of rapeseed with rich genetic variation can be developed through more in-depth and extensive studies by multi-omics combination techniques, such as genomics, transcriptomics, proteomics, metabolomics, and ionomics. Genetic regulatory networks for high N efficiency should be constructed by integrating information of different omics levels, so as to dig out key candidate genes for high N efficiency in rapeseed and elucidate the biological mechanism of its tolerance to low nitrogen.
(3) Mining excellent allelic variation of the key genes for high N efficiency and establishing an efficient molecular design breeding technology system. Currently, mutant materials were mainly used to study the mechanism of N transport that have not been found in rapeseed. Therefore, researchers should strive to explore the excellent allelic variation of key genes controlling N use efficiency under different N sources and different N supply conditions, so as to provide more candidate gene loci for molecular breeding of N efficiency in rapeseed. The way in which to explore the excellent allelic variation and key genes are mainly achieved through the following steps: Firstly, building a widely representative rapeseed natural population. Secondly, identifying the phenotypes related to N efficiency in multi-environment and multi-batch hydroponics, pot, and field experiments of a natural rapeseed population, then combining them with the high-throughput resequencing results to obtain QTLs significantly associated with N efficiency by genome-wide association analysis. Thirdly, carrying out transcriptome, metabolome, and other omics analyses for N efficiency and N inefficiency germplasm. Finally, the N-efficient genes in the QTL region identified above are combined with the differentially expressed genes identified by various omics analysis, the N-efficiency-related candidate genes are screened, the superior haplotype analysis is conducted according to the natural variation of the candidate genes in the population combined with their phenotypes, and the N-efficiency-related candidate genes are determined. It is worth mentioning that some key genes perform their functions under relatively extreme conditions, and thus a large number of studies have been conducted under extreme or low N conditions through hydroponics, potting, or field experiments. In addition, establishing an efficient molecular design breeding technology system and integrating the newly excavated N-efficient genes into excellent backbone parents to breed new N-efficient rapeseed varieties with “less input and more output”. For example, Chu et al. successfully transferred the N-efficient gene *OsNRT1.1B* into Japonica rice, wherein the yield increased by 30–33% under the condition of reducing N application by half, and the NUE also increased by 30% [81].

## Figures and Tables

**Table 1:** Types and characteristics of nitrogen uptake, transfer, and assimilation genes.

Gene Category	Gene Name	Gene ID	Features	References
Nitrate transporters	*BnNRT1.1*	*BnaA06G0084100ZS* *BnaA08G0280200ZS* *BnaA09G0649200ZS* *BnaC05G0104000ZS* *BnaC05G0104100ZS* *BnaC08G0214800ZS* *BnaC08G0508100ZS*	A dual-affinity nitrate transporter. Involved in nitrate signaling, which enables the plant root system to detect and exploit nitrate-rich soil patches.	[51,56]
	*BnNRT1.4*	*BnaA07G0151200ZS* *BnaC04G0211400ZS* *BnaC07G0217200ZS*	Low-affinity nitrate transporter.	[71]
	*BnNRT1.5*	*BnaA05G0324300ZS* *BnaA09G0391300ZS* *BnaC05G0284500ZS* *BnaC05G0339400ZS*	Involved in xylem transport of nitrate from root to shoot.	[57,71]
	*BnNRT1.8*	*BnaA01G0117900ZS* *BnaA03G0461700ZS* *BnaC01G0143800ZS* *BnaC03G0720800ZS* *BnaC07G0437700ZS*	Functions in nitrate removal from the xylem sap.	[57]
	*BnNRT2.4*	*BnaA10G0160100ZS* *BnaC09G0443000ZS*	Member of the high-affinity nitrate transporter family.	[71]
	*BnNRT2.5*	*BnaA08G0276500ZS* *BnaC08G0220400ZS*	Member of the high-affinity nitrate transporter family.	[51]
	*BnNRT2.6*	*BnaA01G0234100ZS* *BnaA01G0234200ZS* *BnaA06G0186600ZS* *BnaA06G0186700ZS* *BnaC01G0301400ZS* *BnaC01G0301600ZS* *BnaC03G0602800ZS* *BnaC03G0603000ZS*	Member of the high-affinity nitrate transporter family.	[51]
	*BnNRT2.7*	*BnaA02G0054200ZS* *BnaC02G0063100ZS*	A nitrate transporter that controls nitrate content in seeds.	[51]
Ammonium transporters	*BnAMT1;1a*	*BnaA05G0145700ZS* *BnaC06G0150000ZS* *BnaC08G0072000ZS*	Encodes a plasma membrane localized ammonium transporter.	[51,71]
	*BnAMT1;2a*	*BnaA09G0017700ZS* *BnaC05G0575800ZS* *BnaC07G0519800ZS* *BnaC09G0000500ZS*	Encodes an ammonium transporter protein believed to act as a high-affinity transporter.	[71]
	*BnAMT1;3a*	*BnaA01G0285300ZS* *BnaA07G0069600ZS* *BnaC01G0349000ZS* *BnaC07G0104500ZS*	Encodes a plasma-membrane-localized ammonium transporter.	[71]
	*BnAMT1;4a*	*BnaA01G0083600ZS* *BnaA03G0506500ZS* *BnaC01G0101600ZS* *BnaC07G0484800ZS*	Ammonium transporter.	[71]
	*BnAMT2;1a* *BnAMT2;1b*	*BnaA04G0246200ZS* *BnaA05G0072900ZS* *BnaC04G0083600ZS* *BnaC04G0561000ZS*	Functional ammonium transporter, constitutively expressed.	[71]
Nitrogen assimilation	*BnGln1;1*	*BnaA04G0096800ZS* *BnaA07G0108800ZS* *BnaC04G0378100ZS* *BnaC04G0386000ZS*	Encodes a cytosolic glutamine synthetase; the enzyme has high affinity with substrate ammonium.	[51,72,73]
	*BnGln1;2*	*BnaA02G0159900ZS* *BnaA03G0181000ZS* *BnaC02G0204200ZS*	Encodes a cytosolic glutamine synthetase; the enzyme has high affinity with substrate ammonium.	[51,72]
	*BnGln1;4*	*BnaA02G0065000ZS* *BnaA10G0200900ZS* *BnaC02G0075000ZS* *BnaC09G0498800ZS*	Encodes a cytosolic glutamine synthetase; the enzyme has high affinity with substrate ammonium.	[51,72,73]
	*BnNR1*	-	Encodes a cytosolic glutamine synthetase; the enzyme has high affinity with substrate ammonium.	[51]
	*BnNR2*	-	Encodes a nitrate reductase structural gene. Involved in nitrate assimilation.	[51]
Transcription factors	*BnWRKY33S*	*BnaA03G0185200ZS* *BnaA04G0247200ZS* *BnaA05G0071200ZS* *BnaC03G0217300ZS* *BnaC04G0080600ZS* *BnaC04G0562300ZS* *BnaC07G0339900ZS*	Member of the plant WRKY transcription factor family. Involved in response to various abiotic stresses, especially salt stress.	[73]
	*BnWRKY70*	*BnaA04G0035900ZS* *BnaA07G0195100ZS* *BnaA09G0519800ZS* *BnaC04G0308100ZS* *BnaC06G0198900ZS* *BnaC08G0362900ZS*	Member of WRKY transcription factor. Functions as an activator of SA-dependent defense genes and a repressor of JA-regulated genes.	[73]
	*BnMYB4*	*BnaA06G0451000ZS* *BnaA08G0200500ZS* *BnaC03G0669700ZS* *BnaC07G0548300ZS*	Encodes a R2R3 MYB protein that is involved in the response to UV-B. It functions as a repressor of target gene expression.	[73]
	*BnMYB44*	*BnaA02G0355600ZS* *BnaA07G0141400ZS* *BnaC02G0479100ZS* *BnaC02G0553100ZS* *BnaC07G0205300ZS*	Member of the R2R3 factor MYB gene family involved in mediating plant responses to a variety of abiotic stimiuli.	[73]
	*BnMYB51*	*BnaA06G0127950ZS* *BnaA08G0256450ZS* *BnaA09G0613050ZS* *BnaC05G0156000ZS* *BnaC08G0246750ZS* *BnaC08G0468450ZS*	Encodes a member of the R2R3-MYB transcription family. Involved in indole glucosinolate biosynthesis.	[73]

## Data Availability

Not applicable.

## Abbreviations

N: nitrogen; *B. napus*: *Brassica napus*; NUE: nitrogen use efficiency; NR: nitrate reductase; NiR: nitrite reductase; GS: glutamine synthetase; GOGAT: glutamate synthetase; NRT: nitrate transporter; AMT: ammonium transporter.
